# Successful pregnancy in a patient with mitochondrial cardiomyopathy due to ACAD9 deficiency

**DOI:** 10.1002/jmd2.12157

**Published:** 2020-09-21

**Authors:** Talia Jacobi‐Polishook, Naama Yosha‐Orpaz, Yair Sagi, Dorit Lev, Tally Lerman‐Sagie

**Affiliations:** ^1^ Pediatrics Department Edith Wolfson Medical Center Holon Israel; ^2^ Metabolic‐Neurogenetic Clinic Edith Wolfson Medical Center Holon Israel; ^3^ Pediatric Neurology Unit Edith Wolfson Medical Center Holon Israel; ^4^ Department of Obstetrics and Gynecology Sourasky Medical Center Tel Aviv Israel; ^5^ Sackler School of Medicine Tel‐Aviv University Tel‐Aviv Israel; ^6^ The Rina Mor Institute of Medical Genetics Edith Wolfson Medical Center Holon Israel

**Keywords:** ACAD9, cardiomyopathy, fatty acid oxidation, mitochondrial disease, pregnancy, riboflavin

## Abstract

Acyl‐CoA dehydrogenase family member 9 (ACAD9) is an enzyme essential for the assembly of mitochondrial respiratory chain complex I. ACAD9 deficiency can cause lactic acidosis, myopathy, cardiomyopathy, intellectual disability, and early demise. We present a patient with mitochondrial myopathy, hypertrophic cardiomyopathy, and epilepsy due to recessive ACAD9 mutations. A muscle biopsy depicted ragged red fibers, and decreased activity of complex I of the respiratory chain. Treatment with riboflavin was initiated at the age of 4 years due to complex I deficiency (before the genetic diagnosis), resulting in symptomatic improvement of the cardiomyopathy, exercise intolerance, and lactate levels. A novel homozygous ACAD9 mutation was found: c.398G>A; p.Ser133Asn at the age of 23 years. Three years later she sustained a normal pregnancy, and gave birth to a healthy baby girl delivered by an elective Cesarean section. To the best of our knowledge, this is the first description of a successful pregnancy and delivery in a patient with this rare mitochondrial disease.

**Synopsis**

Riboflavin‐treated women with a mitochondrial cardiomyopathy/myopathy due to ACAD9 deficiency can have a normal pregnancy and delivery.

## 1 INTRODUCTION

Acyl‐CoA dehydrogenases (ACADs; EC 1.3.99.13) are mitochondrial enzymes that catalyze the initial rate‐limiting step in the beta‐oxidation of fatty acyl‐CoA. ACAD9 belongs to a group of ACADs that act on fatty acids containing 14 to 20 carbons, and also plays a role as an assembly factor for mitochondrial respiratory chain complex I.^1^, ^2^, ^3^ He et al^4^ were the first to establish the clinical phenotype of ACAD9 deficiency (OMIM# 611103), that was later broadened to include: myopathy, exercise intolerance, lactic acidosis, cardiac involvement (cardiomyopathy, heart failure, arrhythmia), central nervous system symptoms (developmental delay, intellectual deficits, seizures, encephalopathy), renal dysfunction, optic atrophy, liver dysfunction, ovarian failure, and death in infancy.^2^, ^5^, ^6^, ^7^

Women with inherited metabolic disorders, including those with previously life‐limiting conditions such as ACAD9 deficiency, are reaching childbearing age more often due to advances in early diagnosis and improved pediatric care. It is commonly assumed that mitochondrial diseases can worsen during pregnancy, but the information in the literature is sparse.^8^, ^9^ Treatment can be challenging and there are no established guidelines. We describe for the first time the management of a successful pregnancy of a patient with ACAD9 deficiency.

## 2 PATIENT AND METHODS

After informed consent, we retrospectively reviewed the patient's files from the age of diagnosis for clinical and biochemical data, treatment regime before and during pregnancy, and obstetrical outcome.

Genomic DNA was extracted from peripheral blood by the Puregene kit (Gentra, Minneapolis, Minnesota), according to the manufactures' instructions.

Sequencing of *ACAD9* (NM_014049.4) was carried out on NextSeq500 (Illumina, San Diego, California) as 100‐bp paired‐end runs, average depth 100×. Image analysis and base calling were performed with the Genome Analyzer Pipeline version 1.5 with default parameters. Reads were mapped to the human reference genome sequence (assembly GRCh37/hg19) using the Burrows‐Wheeler Alignment Tool (BWA), and allelic variants were analyzed using Variant Studio (Illumina) and IGV.

Sanger confirmation of the *ACAD9* mutation in the patient and parents was performed using the following primers: F‐5′‐ccggcctgtattgtgatttt and R‐5′ tatgcctccatgtgactcca.

## 3 CASE REPORT

The patient was a product of a normal pregnancy and delivery of nonconsanguineous Iraqi Jewish parents. Motor milestones were delayed, while language development was premature. Exercise intolerance accompanied by leg pain became prominent after initiation of independent walking.

Family history was significant for an older brother with a hypertrophic cardiomyopathy and lactic acidosis (diagnosed at the age of 3 years), who died suddenly at the age of 9 years after a minor blunt chest trauma. Two other siblings suffered from neurosensory hearing loss.

She presented at the age of 4 years due to muscle weakness and exercise intolerance. Creatine phosphokinase (CPK) was 79 IU/L (20‐180), lactate was 54 mg/dL (9‐22 mg/dL). A metabolic evaluation, including urinary organic acids, blood amino acids, carnitine, and ammonia was normal.

An echocardiogram demonstrated a hypertrophic cardiomyopathy. A muscle biopsy revealed ragged red fibers and decreased Complex I activity. Electron microscopy showed increased number of mitochondria with changes in their structure and size. The patient was diagnosed with a mitochondrial myopathy and cardiomyopathy. Sequencing of the mitochondrial DNA did not disclose any mutations known to cause myopathy, cardiomyopathy nor deafness.

Following the diagnosis of a mitochondrial disorder due to complex I deficiency, treatment with coenzyme Q, carnitine, antioxidants (vitamins A, E, C, selenium) and riboflavin 100 mg/day was initiated. There was gradual improvement in lactate levels, exercise intolerance, muscle strength, and cardiomyopathy. Furthermore, the echocardiogram normalized. At the age of 5 years and 4 months nonsustained ventricular tachycardia was diagnosed on Holter electrocardiography (ECG), and treatment with amiodarone was started. At the age of 10 years amiodarone was stopped and the Holter ECG remained normal.

At the age of 21 years she sustained a nocturnal seizure. An electroencephalogram (EEG) was normal. Brain computed tomography showed ventricular asymmetry. An ECG showed sinus rhythm with T wave inversion in the inferior and lateral walls. An echocardiogram showed mild hypertrophy of the lateral wall of the left ventricle with normal function. A stress echocardiogram was normal. A cardiopulmonary exercise test revealed that the complaint of exercise intolerance was probably the result of quick transfer to anaerobic activity due to the mitochondrial disorder, rather than a primary cardiac abnormality. Magnetic Resonance Imaging (MRI) of the heart showed increased T2 signal in the mediolateral ventricular wall and apex, and late patchy enhancement in the lateral and medial walls. Based on the cardiac MRI findings, a past history of arrythmia, and the sudden death of the patient's brother, a cardiac event was assumed, and a defibrillator was implanted. Two weeks later the defibrillator was activated twice due to atrial tachycardia with a rate of 200 to 260 beats/min. An ablation attempt was not successful, and treatment with metoprolol was started. Nocturnal events recurred and were diagnosed based on the semiology as focal to bilateral seizures. Despite levetiracetam treatment seizures recurred. Sulthiame was added with complete control. Sulthiame and levetiracetam were successfully replaced by lamotrigine in anticipation of a future pregnancy. Following the completion of a BA in education and an MA in educational counseling she worked as a teacher.

After the description in the literature of *ACAD9* related riboflavin responsive cardiomyopathy the gene was sequenced and a homozygous mutation was found—a change in exon 4: C.398G>A; p.Ser133Asn. This variant had not been previously described and was predicted as pathogenic according to the prediction software Mutation Taster. Both parents were heterozygote carriers.

### 3.1 Pregnancy

At the age of 26 years she became pregnant. She continued medical treatment with metoprolol and lamotrigine. Riboflavin dose was increased to100 mg X3/day. She was tightly followed all through the pregnancy by a multidisciplinary team including obstetrics, cardiology and mitochondrial disease expert.

The course was uneventful until the 38th week of gestation. Although she complained more of exercise intolerance, manifesting as fatigue and leg pain after prolonged activity. Her daily function did not change and she continued her work as a teacher. Echocardiography showed good heart function, normal ventricular size, left ventricle (LV) wall thickness, global systolic function (EF 60%), LV diastolic function and pulmonary artery pressure. There was mild left atrial dilatation and tricuspid regurgitation. A 24 hours ECG loop recorder did not depict any signs of arrhythmia. Her epilepsy was well controlled with lamotrigine. Neurological exam including muscle strength were normal. Lactate levels remained as before pregnancy between 31 and 34 mg/dL. CPK was normal—44 to 45 U/L. The fetal anatomy surveys at 15, 22, and 32 weeks of gestation were unremarkable.

At 36 weeks of gestation a multidisciplinary discussion was held regarding the mode of delivery including specialists in obstetrics, maternal‐fetal medicine, metabolic diseases, neurology, and anesthesiology. A literature search did not reveal any description of a pregnancy in a woman with ACAD9 deficiency. There was scarce information regarding delivery of women with mitochondrial disorders.^8^, ^9^ We assumed that prolonged labor could result in an energetic crisis and thus endanger the patient and fetus and therefore an elective Cesarean‐section was planned at 39 weeks.

At 38 weeks of gestation following an ultrasound that demonstrated growth retardation (estimated weight at the fifth percentile) and oligohydramnios (amniotic fluid index of 5.7) it was decided to advance the delivery. A Cesarean section with spinal anesthesia, using bupivacaine, was performed. During the delivery she received intravenous fluids with glucose 5% and normal saline, and was hemodynamically stable. A healthy baby girl was born weighing 2130 g (−2.5 SD), with an Apgar scores of 9 and 10 at 1 and 5 minutes, respectively. The postnatal period was uneventful. The mother and child were discharged on time.

The patient's postoperative course was unremarkable, without any signs of metabolic crisis or acidemia. She was discharged home on her fifth postoperative day.

## 4 DISCUSSION

There is insufficient information regarding management and outcome of pregnancies of women with mitochondrial disorders. There is a constant concern that the increased metabolic demands may exacerbate symptoms during pregnancy and a prolonged or strenuous labor might endanger the mother by inducing energy failure. Karaa et al^8^ in a retrospective study describe the obstetric history of 103 women (370 pregnancies) with mitochondrial diseases or dysfunctions. Many mitochondrial symptoms aggravated or first appeared during pregnancy: fatigue, exercise intolerance, nausea, cardiovascular related symptoms, constipation, cramps, seizures, headache, muscle weakness, and neuropathy. Autonomic dysfunction symptoms seem to flare. Obstetric complications were frequent: vaginal bleeding, proteinuria, new onset high blood pressure, gestational diabetes, small for gestational age fetuses, oligo or polyhydramnios and pre‐eclampsia. Only 29% did not have gestational complications. Twenty‐nine percentage of the pregnancies ended in miscarriages and 12% in preterm labor. In 68%, a vaginal delivery was achieved. Most neonates were full term. The babies tended to have more neonatal complications and congenital anomalies (mostly cardiac).

In another review of 10 pregnant women with mitochondrial diseases, the most common obstetric complications were preeclampsia and threatened preterm labor.^9^ The pregnancy had diverse effects: serious symptoms such as symptomatic Wolff‐Parkinson‐White syndrome, paraesthesias, and focal segmental glomerulosclerosis, or mild symptoms such as exercise intolerance or muscle weakness which resolved postnatally. Three remained asymptomatic.

The recommendations for management of women with mitochondrial diseases during pregnancy include: evaluation and monitoring by a maternal‐fetal specialist in consultation with a mitochondrial disease expert, careful management of increased mitochondrial symptoms and signs, peripartum fluid and caloric support, adjustment of medications and supplements, and screening for congenital anomalies.^8^

We report the first description of a successful pregnancy of a woman with mitochondrial cardiomyopathy and myopathy related to *ACAD9*. Most of the historical cases with ACAD9 deficiency including our patient's brother died in early childhood.^5^ Due to early riboflavin treatment our patient achieved adulthood and is now 29 years old.

Early symptoms included hypotonia, delayed motor milestones, muscle weakness, exercise intolerance, cardiac hypertrophy and elevated lactate levels. Following the empiric initiation of riboflavin treatment (25 years ago) there was an improvement in the cardiomyopathy and lactate levels.

Riboflavin is the vitamin precursor of flavin adenine dinucleotide (FAD), which is the cofactor of ACADs.^10^ Haack et al^10^ tested in 2010 the impact of riboflavin supplementation on mutant cell cultures from two patients with ACAD9 mutations and found a significant increase in complex I activity. Repp et al^5^ reported patients with predominant myopathic features who had alleviation of symptoms with riboflavin treatment. There was a significant increase in survival in the treated group.

Our patient was carefully followed and monitored during pregnancy according to the recommendations suggested by Karaa et al.^8^ The riboflavin dose was increased. She remained in good health during gestation with only a mild increase in exercise intolerance. Her echocardiography showed good heart function. She did not have any signs of arrhythmia, and epilepsy was well controlled. However, towards the end of gestation intrauterine growth restriction and oligohydramnios were observed. A decision to deliver by Cesarean section was made in order to reduce the risk of an energy crisis. As a precaution, intravenous fluids with glucose 5% were administered. A healthy baby girl was born.

## 5 CONCLUSION

Pregnancy of riboflavin‐treated women with mitochondrial cardiomyopathy/myopathy due to ACAD9 deficiency can be uneventful and result in delivery of a normal child.

### AUTHOR CONTRIBUTIONS

Talia Jacobi‐Polishook was involved in analysis and interpretation of data, drafting the manuscript, reviewing the patient's files, collecting clinical, and biochemical data. Naama Yosha‐Orpaz was involved in analysis and interpretation of data, and revising the article critically for important intellectual content. Yair Sagi was involved in analysis and interpretation of data, and revising the article critically for important intellectual content, clinical management of the patient during pregnancy. Dorit Lev was involved in analysis and interpretation of data, genetic analysis, clinical management of the patient, and revising the article critically for important intellectual content. Tally Lerman‐Sagie was involved in analysis and interpretation of data, revising the article critically for important intellectual content, clinical management of the patient from early childhood, and guarantor for the article.

## CONFLICT OF INTEREST

The authors declare no conflicts of interest.

## ETHICS STATEMENT

Ethics approval was not required for publication of this case report.

## INFORMED CONSENT

We confirm that no patient identifiable information has been included in the manuscript. Informed consent was obtained from the patient included in the study.

## ANIMAL RIGHTS

This article does not contain any studies with animals.
